# On the Buffy Coat of the Blood during Inflammation, in Answer to Mr. Lanyon

**Published:** 1819-03

**Authors:** 


*For the London Medical and Physical Journal.*

*On the Buffy Coat of the Blood during Inflammation, in Answer to Mr. Lanyon;* by a Member of the Royal College.

I FEEL much diffidence in trespassing upon the important pages of the Medical and Physical Journal, in order to elucidate the advantages derived from using several vessels to receive the blood, in blood-letting in inflammatory diseases.

Mr. Lanyon should have consulted authors before he put the question to the profession, and he would have found that, in all inflammatory diseases, the first-drawn blood scarcely ever shows any of the buffy coat, unless a very considerable portion is abstracted at once in a single vessel. I invariably adopt a practice, which I have ever considered too general to need publication, of permitting the blood to deposit in several vessels (tea-cups are the most proper), by which I have been better enabled to distinguish the stage or extent of inflammation. We never can draw a fair inference by allowing the blood to flow into a single vessel; for the flow of the blood at the commencement is generally sluggish and tardy, which allows it to coagulate very speedily, thus entangling those particles which afterwards swim upon the surface of the crassamentum, constituting this peculiarity, or buffy coat: presently the pulse, which before was labouring, and the arterial system much oppressed, rises from under the load of the constitution, the circulation is increased, and the jet of blood becomes uniform and steady. Now time has been allowed for the viscid and heterogenous ingredients to be more intimately united, the deposition is permitted to form gradually, and of course the buffy coat of the blood exhibits itself. This is constantly to be observed in bleeding patients during inflammatory stages of disease.

The blood may still be "homogenous throughout," notwithstanding Mr. Lanyon's accident "would seem to argue the contrary;" for he may, with the utmost decorum, divest himself of entering into "idle speculations or useless hypothesis," by merely bringing to his recollection the laborious state of the pulse and consequent general depression, which never fail of being relieved during the flow of the blood. It would be a curious speculation, and probably not without some interest, to investigate the phenomena of the powers of life during venesection: it has been a subject commenced, but with very little advancement, owing probably to the inconvenience the subject of experiment, as well as the experimenter himself, would be exposed to.

*Suffolk-street, London;*
*February* 6, 1819.

---

*For the London Medical and Physical Journal.*

*Examination of Mr. Samuel Young's Remarks on Mr. Astley Cooper's Operation of tying the Aorta;* by C. R.

